# Artificial intelligence and robotics in TKA surgery: promising options for improved outcomes?

**DOI:** 10.1007/s00167-022-07035-x

**Published:** 2022-06-20

**Authors:** Rüdiger von Eisenhart-Rothe, Florian Hinterwimmer, Heiko Graichen, Michael T. Hirschmann

**Affiliations:** 1 Department of Orthopaedics and Sports Orthopaedics, Klinikum Rechts der Isar, Technical University Munich, Ismaningerstr. 22, 81675 Munich, Germany; 2 Department of Arthroplasty, Sports Medicine and Traumatology, Orthopaedic Hospital Lindenlohe, Lindenlohe 18, 92421 Schwandorf, Germany; 3 Department of Orthopaedic Surgery and Traumatology, Kantonsspital Baselland (Bruderholz, Liestal, Laufen), 4101 Bruderholz, Switzerland; 4 Institute for AI and Informatics in Medicine, Technical University of Munich, Munich, Germany

Digitalisation has entered Orthopaedics in the last decade, and is meanwhile a relevant part of pre-, intra-, and postoperative processes. Thereby, the transformation away from an experience-based to a data-based more patient-specific treatment is the basis for an extensive analysis of various parameters and its interpretation regarding their relevance during the treatment pathway. In different fields of daily life, artificial intelligence (AI) and, especially, machine learning (ML) have shown to use, analyse, and interpret enormous amount of data and based on intelligent algorithms to improve finally quality along the process.

Machine learning represents a distinct application of AI, which describes algorithms for automatic and incremental function optimization. This can be used to make predictions by detecting non-linear relationships in large data sets [5]. Both offer tremendous new possibilities and clearly are promising options for the field of orthopaedic surgery, and in particular for total knee arthroplasty (TKA). Here, patients were treated in a standardized manner over long periods of time with the goal of achieving the same alignment while neglecting individual or personal variations in alignment and morphology variations [3]. While the goal was always the same, the individual knees and especially the alignment and shape were not. From today's perspective, you do not have to be a rocket scientist to understand that due to the variability of anatomy, personalized targeting is the future. Therefore, many knee surgeons strive to personalize their alignment goals, but without knowing the consequences of the changes made. The safe transition from a purely standard mechanical alignment to a more personalized alignment might be a rocky road [13]. To make the road a less rocky or paved one, it is of utmost importance to have support from state-of-the-art data analysis tools to answer pertinent questions such as [12]: which is the best alignment goal for which phenotype? What are the phenotypic clinical and functional outcomes after different types of personalized alignment strategies?

Finally, having a tool supporting orthopaedic surgeons before any therapy is initiated, giving them the information, which treatment option or alignment strategy might be the most promising for a specific patient, would hopefully help to minimize the rate of the reported 20% unhappy patients after TKA. Moreover, the effect of further outcome-relevant parameters such as patient expectation, BMI, or compliance can be calculated and provide insight into their potential non-linear relation.

Over the past decades, TKA surgery has strived for more and more precision to achieve a standard goal that is the same for all patients. In recent years, however, it was realised that our patient population not only implicates a wide variety of individual preoperative factors (e.g., activity levels, mental status, knee pathologies), but also various different anatomical knee parameters (bone morphology, ligamentous and soft-tissue morphology) [1, 2, 6, 7, 9]. This variation, which has not yet been fully understood, has recently led to a more individualized approach to treatment. However, the sheer quantity and interplay of parameters makes it difficult to understand which factor has which influence on which part of the result. Such complex analysis can only be performed with the help of AI and ML.

Until now, only a few studies have been published using ML for prediction of complications, costs and outcome. The majority of these studies failed to show a potential of ML in TKA surgery [5]. However, a few have been able to show that at least some aspects of this vast field of research can be adequately evaluated and predictions can be made for specific situations with specific data [4, 10, 11]. This situation leads to the overall challenge of AI and ML in orthopaedics: only by assessing all relevant factors correctly, the algorithms will be able to detect their concrete relevance. Even though some algorithms have been developed to deal with a very limited amount of missing data, the absence of important parameters in the analysis can have a substantial impact on the result. In addition, all inaccuracies should ideally be considered. Second, validity must be ensured through accurate data documentation in the first instance and possibly through subsequent data screening and cleaning. And finally, the data must originally contain all relevant information, so that, subsequently, complex relationships can be derived. Thus, large data sets with sufficient data depth and width as well as quality are required. In orthopaedics, this involves a large number of patients and the monitoring of a large number of parameters, whereas some of the relevant parameters are still unknown.

To gain such huge data sets, it is required to focus more on multicentre studies as a promising option. However, data privacy as well as information security are of highest priority, especially when working with sensitive patient data. For research purposes though, these paradigms often present obstacles for data exchange and analysis. Therefore, the infrastructures in the research institutions must be further developed to meet today's requirements. One technique for leveraging state-of-the-art IT-infrastructure for AI needs is federated learning, where a single model is trained on multiple servers, so that the original data do not have to leave the research facility [8].

Another promising option for analysis might be the different arthroplasty registries; nonetheless, the parameters included in most of the national registries are very limited. In particular, if the influence of alignment philosophies on outcome is to be understood, all relevant parameters must be retrieved for each patient. This includes functional parameters such as fixed flexion deformity or hyperextension/laxity; HKA, bony morphology (MPTA, LDFA), and also the gap differences throughout the entire range of motion as well as cartilage thickness left on all six cartilage areas. All those parameters can only be stored by digital OR tools, such as navigation or robotics.

The introduction of robotics in TKA promises the perfect execution of the surgical plan and reporting of the achieved intraoperative result. In addition, due to a better understanding of intraoperative gap geometry, the path of personalized alignment is secured.

However, intraoperative data storage is only one part of data which should be collected. Data collection of pre- and postoperative data are at least as crucial. Therefore, solutions that enable data acquisition and integration of different platforms such as patient information systems, PACS, etc. urgently are needed.

In conclusion, AI and ML in combination with navigation or robotics are promising options for answering a long list of complex treatment questions of severe TKA such as: which patients will benefit from TKA or which subtype should not receive surgery? Which knee will benefit from which alignment strategy most? After all, before meaningful answers on all those questions can be received, human brains must work together as a group to define all parameters, so that the machine then can do its part. In addition, the digital infrastructure of hospitals must enable easy data collection from the various sources within the hospital, but also, and especially, between different hospitals.
